# Supplementary material for: Autonomous Sensory Meridian Response (ASMR): a flow-like mental state
Source: PeerJ. 2015 Mar 26;3:e851. doi: 10.7717/peerj.851 (PMC4380153; doi:10.7717/peerj.851)
Supplement: Appendix S1 [file peerj-03-851-s001.docx]

*Autonomous Sensory Meridian Response (ASMR) questionnaire*

**Are you…**

□ Male □ Female □ Non-binary/other

**What is your age?**

*[drop list of numbers]*

**Where are you located?**

*[drop list of locations]*

**Do you suffer from any chronic pain or illness?**

□ Yes □No

**Please specify.**

***Synaesthesia is defined as perception in one sense triggering sensation in another, unstimulated sense. For example, you may ‘see’ the letters as having colours, or sense shapes from music. For further elaboration, see here: http://www.uksynaesthesia.com/whatis.html***

**Do you have any type of synaesthesia?**

□ Yes □No

**If so, please elaborate.**

**Do you take any medications?**

□ Yes □No

Please specify which medications you take.

**Do you watch ASMR videos?**

□ Yes □No

**How many ASMR videos do you typically watch in a single session?**

[drop list of numbers]

**What time of day do you usually watch ASMR videos?**

- Upon Waking
- Mid-morning
- Mid-day
- Afternoon
- Evening
- Before sleeping
- Whenever I have spare time

**Do you require specific conditions to achieve ASMR?**

□ Yes □No

**Please briefly describe the conditions you require to achieve ASMR sensations (e.g. busy room, bright lighting, etc.)**

**Do you feel a tingling sensation when watching ASMR videos?**

□ Yes □No

**Are these tingling sensations triggered by specific stimuli?**

□ Yes □No

**Please tick all the items that trigger your tingling sensations while viewing ASMR videos.**

- Crisp sounds (e.g. tapping, crinkling plastic)
- Whispering
- Water pouring
- Personal attention (e.g. face touching)
- Vacuuming
- Aeroplane noise
- Laughing a lot and doing all the things that make you happy
- Smiling
- Watching repetitive tasks (e.g. towel folding)
- Slow movements
- A specific combination of two or more of these options (please elaborate)
- Other (please elaborate)

**Please briefly elaborate on your selection of 'A specific combination of two or more of these options'.**

**Please briefly describe any 'other' triggers. Please separate triggers by commas (e.g. tickling, dog walking, trainspotting...).**

**Do any stimuli stop or prevent this tingling sensation from continuing?**

□ Yes □No

**What stimuli will stop any tingling sensation you are feeling? Please be brief and use as few words as possible.**

**Does this tingling sensation always originate in one area of your body?**

□ Yes □No

**Where can your tingles originate?**

- Head
- Shoulders
- Chest
- Back
- Arms
- Stomach/lower abdomen
- Genitals
- Hips
- Legs
- Feet

**Do you feel this tingling sensation more on one side of your body than the other?**

□ Yes, left □ Yes, right. □ No, both sides are the same.

**Does the intensity of these tingles vary from session to session of ASMR video viewing?**

□ Yes □No

**Does the intensity of these tingles vary at different times during a session of ASMR video viewing?**

□ Yes □No

**What increases the intensity of the tingling sensation?**

- Sounds that I like
- Visuals that I like
- Visuals I don’t like
- Visuals I don’t expect
- Sounds I don’t like
- Sounds I don’t expect
- Other

**Does more intense tingling result in the sensation moving to other areas of your body?**

□ Yes □No

**Please tick the body areas that experience tingles when the sensation is most intense.**

- Head
- Shoulders
- Chest
- Back
- Arms
- Stomach/lower abdomen
- Genitals
- Hips
- Legs
- Feet

**How long does the tingling sensation normally last?**

**Is the tingling sensation triggered more easily by hearing in one ear than the other?**

□Yes, my left ear. □ Yes, my right ear. □ No, both ears are the same.

**Does your medication affect your experience of tingling at all?**

□ Yes □No

**At what age did you first experience this tingling sensation?**

**Do you have any family members that experience ASMR?**

□ Yes □ No □ Unsure/I’ve never asked

**Are your tingling sensations/ASMR triggered by anything other than online videos?**

□ Yes □ No

**What other stimuli trigger your tingling sensations/ASMR? Please describe briefly.**

**Please rate the following statements in terms of how true they are for you while experiencing tingles.**

- My attention is focused entirely on what I am watching.

| Not my experience at all  1 | 2 | 3 | 4 | Completely representative of my experience  5 |
| --- | --- | --- | --- | --- |
| ○ | ○ | ○ | ○ | ○ |

- My attention is focused entirely on what I am feeling.

| Not my experience at all  1 | 2 | 3 | 4 | Completely representative of my experience  5 |
| --- | --- | --- | --- | --- |
| ○ | ○ | ○ | ○ | ○ |

- Time seems to alter (slow down or speed up).

| Not my experience at all  1 | 2 | 3 | 4 | Completely representative of my experience  5 |
| --- | --- | --- | --- | --- |
| ○ | ○ | ○ | ○ | ○ |

- Things seem to happen automatically.

| Not my experience at all  1 | 2 | 3 | 4 | Completely representative of my experience  5 |
| --- | --- | --- | --- | --- |
| ○ | ○ | ○ | ○ | ○ |

- It is no effort to keep my mind on what is happening.

| Not my experience at all  1 | 2 | 3 | 4 | Completely representative of my experience  5 |
| --- | --- | --- | --- | --- |
| ○ | ○ | ○ | ○ | ○ |

- I feel totally in control.

| Not my experience at all  1 | 2 | 3 | 4 | Completely representative of my experience  5 |
| --- | --- | --- | --- | --- |
| ○ | ○ | ○ | ○ | ○ |

- Time seems to stop.

| Not my experience at all  1 | 2 | 3 | 4 | Completely representative of my experience  5 |
| --- | --- | --- | --- | --- |
| ○ | ○ | ○ | ○ | ○ |

- I am not worried about what people think of me.

| Not my experience at all  1 | 2 | 3 | 4 | Completely representative of my experience  5 |
| --- | --- | --- | --- | --- |
| ○ | ○ | ○ | ○ | ○ |

**Do you feel that watching ASMR videos has an effect on your mood?**

□ Yes □ No

**Please select the statement that most applies to you.**

□ I feel a change in my mood only when I experience tingling.

□ I feel a change in my mood whether or not I experience tingling.

□ I feel no change in my mood during ASMR.

**How would you rate your mood in these instances? (0 = terrible, the / worst I've ever felt, 100 = Euphoric, the best I've ever felt)**

- My mood just before I watch ASMR videos

| ▼ | | | | |
| --- | --- | --- | --- | --- |
| 0 | 25 | 50 | 75 | 100 |

- My mood during a successful ASMR video session

| ▼ | | | | |
| --- | --- | --- | --- | --- |
| 0 | 25 | 50 | 75 | 100 |

- My mood when I finish a successful ASMR video session

| ▼ | | | | |
| --- | --- | --- | --- | --- |
| 0 | 25 | 50 | 75 | 100 |

- My mood 30 minutes after a successful ASMR session

| ▼ | | | | |
| --- | --- | --- | --- | --- |
| 0 | 25 | 50 | 75 | 100 |

- My mood an hour after a successful ASMR video session

| ▼ | | | | |
| --- | --- | --- | --- | --- |
| 0 | 25 | 50 | 75 | 100 |

- My mood 3 hours after a successful ASMR session

| ▼ | | | | |
| --- | --- | --- | --- | --- |
| 0 | 25 | 50 | 75 | 100 |

- My mood the day after a successful ASMR video session

| ▼ | | | | |
| --- | --- | --- | --- | --- |
| 0 | 25 | 50 | 75 | 100 |

- My mood generally during day to day life

| ▼ | | | | |
| --- | --- | --- | --- | --- |
| 0 | 25 | 50 | 75 | 100 |

**Do you feel that watching ASMR videos had an effect on your symptoms of chronic illness or pain?**

□ Yes □ No

**Please select the statement that most applies to you.**

□ I feel a change in my symptoms only when I experience tingling.

□ I feel a change in my symptoms whether or not I experience tingling.

□ I feel no change in my symptoms during ASMR.

**PLEASE ANSWER ONLY IF YOU SUFFER FROM SYMPTOMS OF CHRONIC ILLNESS OR PAIN. How would you rate the intensity of your symptoms of chronic illness or pain at these times? 0 = No pain, I wouldn't know I had a condition, 100 = The worst I have ever felt my symptoms.**

- Intensity of chronic illness/pain just before I watch ASMR videos.

| ▼ | | | | |
| --- | --- | --- | --- | --- |
| 0 | 25 | 50 | 75 | 100 |

- Intensity of chronic illness/pain during an ASMR video session.

| ▼ | | | | |
| --- | --- | --- | --- | --- |
| 0 | 25 | 50 | 75 | 100 |

- Intensity of chronic illness/pain just after a successful ASMR video session.

| ▼ | | | | |
| --- | --- | --- | --- | --- |
| 0 | 25 | 50 | 75 | 100 |

- Intensity of chronic illness/pain an hour after a successful ASMR video session.

| ▼ | | | | |
| --- | --- | --- | --- | --- |
| 0 | 25 | 50 | 75 | 100 |

- Intensity of chronic illness/pain the day after a successful ASMR video session.

| ▼ | | | | |
| --- | --- | --- | --- | --- |
| 0 | 25 | 50 | 75 | 100 |

- Intensity of chronic illness/pain during day to day life.

| ▼ | | | | |
| --- | --- | --- | --- | --- |
| 0 | 25 | 50 | 75 | 100 |

**Do you feel that watching ASMR videos helps with your sleeping issues?**

□ Yes □ No

**Please select the statement that most applies to you.**

□ I feel a change in my symptoms only when I experience tingling.

□ I feel a change in my symptoms whether or not I experience tingling.

□ I feel no change in my symptoms during ASMR.

**Please rate the following statements in terms of how well you feel they apply to you and your experience of watching ASMR videos.**

- I watch ASMR videos to relieve negative mood.

| Not my experience at all  1 | 2 | 3 | 4 | Completely representative of my experience  5 |
| --- | --- | --- | --- | --- |
| ○ | ○ | ○ | ○ | ○ |

- I don't know why I watch ASMR videos.

| Not my experience at all  1 | 2 | 3 | 4 | Completely representative of my experience  5 |
| --- | --- | --- | --- | --- |
| ○ | ○ | ○ | ○ | ○ |

- I enjoy ASMR videos.

| Not my experience at all  1 | 2 | 3 | 4 | Completely representative of my experience  5 |
| --- | --- | --- | --- | --- |
| ○ | ○ | ○ | ○ | ○ |

- I know what triggers my ASMR.

| Not my experience at all  1 | 2 | 3 | 4 | Completely representative of my experience  5 |
| --- | --- | --- | --- | --- |
| ○ | ○ | ○ | ○ | ○ |

- I enjoy the content of ASMR videos even without tingles.

| Not my experience at all  1 | 2 | 3 | 4 | Completely representative of my experience  5 |
| --- | --- | --- | --- | --- |
| ○ | ○ | ○ | ○ | ○ |

- I watch ASMR videos to relax.

| Not my experience at all  1 | 2 | 3 | 4 | Completely representative of my experience  5 |
| --- | --- | --- | --- | --- |
| ○ | ○ | ○ | ○ | ○ |

- I watch ASMR videos to deal with anxiety.

| Not my experience at all  1 | 2 | 3 | 4 | Completely representative of my experience  5 |
| --- | --- | --- | --- | --- |
| ○ | ○ | ○ | ○ | ○ |

- I watch ASMR videos to deal with stress.

| Not my experience at all  1 | 2 | 3 | 4 | Completely representative of my experience  5 |
| --- | --- | --- | --- | --- |
| ○ | ○ | ○ | ○ | ○ |

- I watch ASMR videos to help me sleep.

| Not my experience at all  1 | 2 | 3 | 4 | Completely representative of my experience  5 |
| --- | --- | --- | --- | --- |
| ○ | ○ | ○ | ○ | ○ |

- I watch ASMR videos as a hobby.

| Not my experience at all  1 | 2 | 3 | 4 | Completely representative of my experience  5 |
| --- | --- | --- | --- | --- |
| ○ | ○ | ○ | ○ | ○ |

- I watch ASMR videos to help me focus.

| Not my experience at all  1 | 2 | 3 | 4 | Completely representative of my experience  5 |
| --- | --- | --- | --- | --- |
| ○ | ○ | ○ | ○ | ○ |

- I watch ASMR videos to ease chronic physical pain.

| Not my experience at all  1 | 2 | 3 | 4 | Completely representative of my experience  5 |
| --- | --- | --- | --- | --- |
| ○ | ○ | ○ | ○ | ○ |

- I watch ASMR videos for sexual stimulation.

| Not my experience at all  1 | 2 | 3 | 4 | Completely representative of my experience  5 |
| --- | --- | --- | --- | --- |
| ○ | ○ | ○ | ○ | ○ |

- I watch ASMR videos to help with a mental health issue other than depression or anxiety.

| Not my experience at all  1 | 2 | 3 | 4 | Completely representative of my experience  5 |
| --- | --- | --- | --- | --- |
| ○ | ○ | ○ | ○ | ○ |

- I watch ASMR videos to help with depression.

| Not my experience at all  1 | 2 | 3 | 4 | Completely representative of my experience  5 |
| --- | --- | --- | --- | --- |
| ○ | ○ | ○ | ○ | ○ |
