# Supplementary material for: Autonomous Sensory Meridian Response (ASMR): a flow-like mental state
Source: PeerJ. 2015 Mar 26;3:e851. doi: 10.7717/peerj.851 (PMC4380153; doi:10.7717/peerj.851)
Supplement: Supplemental Information 3 [file peerj-03-851-s003.docx]

*Follow up of those who reported experiencing synaesthesia*

Original e-mail

Hi there!

A few weeks ago you took part in a questionnaire for us here at Swansea University looking at ASMR. We’ve gone through the collected data, and found your responses to be of particular interest to the study. In particular, your report of experiencing synaesthesia.

I was wondering if you might help us to assess the strength and kind of synaesthesia you have. This data would be of real interest to the scientific community, so if you’d be willing to contribute by briefly providing the extra information listed below, we would be extremely grateful.

**We would like you to very briefly list 4 things (fewer will do, if you can’t think of or don’t have 4) that you experience as synaesthesia.** For example: “I see the letter A as red.” or “I sense downwards movement when I hear violins”

**Please feel free to make any elaborations you need to in a paragraph following your 4 short sentences.**

If you don’t wish to participate in this part of the study, please just ignore this e-mail. Thank you again for helping, it is deeply appreciated.

All the best,

Emma Barratt

Swansea University

Responses

**1. J.L.**

*Grapheme-colour. Colour-emotion. Colour-personality.*

1. All numbers appear in my mind with a corresponding specific color.

2. All letters appear in my mind with a corresponding specific color.

3. Shapes appear as the color associated with the number of lines it has.

4. Many colors are associated with emotions and even distinctive personality traits.

Seeing a mismatch, such as someone writing the letter "Z" as blue, makes me uncomfortable. It's always been like that for me. If I see the alphabet or math problem written out in all black or all blue, this is fine and doesn't bother me. It's only when someone tries to make a "rainbow" pattern and it doesn't correspond with my color associations. Most of my synesthesia is related to colors. There may be other aspects of it I experience everyday that I'm unaware are abnormal. If you have any other questions, I'd be happy to answer.

**2. E.P.**

*Grapheme-colour.*

I get the pulse when I hear noises of genuine happiness, joy,  etc. If someone is faking (fibbing) a reaction to let's say a present, it has no effect.

If the capital letter A is a colour I would say it is red , B is blue, C is yellow. But I don't see letters in colour, nor can I taste sounds or sights (or I don't think I can).

When I bond with something say a dog/puppy for the first few weeks there breath makes the inside of my head tickle like mad. I'm not allergic, I have three rescues. Even other peoples dogs will sometimes trigger it.

**3. C.Z.**

*Sound-movement.*

I feel the notion movement while listening to certain asmr sound montages wich are difficult to describe, but pretty much follow the line of youtube user AirLight.

Both feelings come in form of flashbacks from an early experience I had with LSD in my youth, wich of course I'm not proud to confess, but I tought might be relevant to point out.

**4. H.S.**

*Sound-colour.*

I'd like to preface this by noting that my synesthesia, such as it is, is not particularly strong. Primarily I find it manifests with music, where I see individual synth/instrument sounds as images, thought not particularly coherent ones.

If I am listening to a complex track with many harmonies, then I find I am more likely to experience the sensation, and the image will be correspondingly more complex.

It's interesting to note that both my ASMR and synesthesia are greatly heightened by smoking cannabis, to the point that I have experienced epic visions of entirely fictional events, purely based on the music I am hearing.

**5. J.F.**

*Calendar-space. Number-space.*

In answer to your question, here are 4 examples:

1. When thinking of the days of the week, I “see” them as a oval pattern with the weekend days at the bottom

2. When thinking of years, both forward and backward I see them as a string with both horizontal and vertical components

3. When thinking of regular numbers, similar to number 2, but more vertical and with curves

4. When thinking of the yearly calendar I see them as a more vertical oval with beginning months on the right.

My synaesthesia tends to be most pronounced in aspects of time and numerical instances.

**6. N.H.**

*Grapheme-smell*

1. WHEN I HEAR PEOPLE WALKING ON PAVEMENTS OR TILED FLOORS IN SHOES THAT MAKE TAPPING SOUNDS I GET AN OVERWHELMING FEELING I HAVE TO LAY DOWN AND SLEEP

2. WHEN I READ BOOKS I CAN SMELL THE DRINK DANDELION AND BURDOCK....SO BOOKS =DANDELION AND BURDOCK :)

3. WHEN I SEE THE NUMBER 4.....MY LUCKY NUMBER...I SMELL CHOCOLATE

**7. B.N.**

*Number-colour. Grapheme-colour.*

Ever since I was little, I've seen numbers as certain colors. Most letters can be associate with colors as well. They're consistent, too - it's kind of weird! Just a few examples: five is orange, six is green, seven is red, E is yellow, B is blue, Q is green. Hopefully this helps!

**8. C.G.**

*Number-colour. Number-personality. Word-personality. Meaning-sound(?).*

1. Numbers, to me, have a distinct color when I picture them in my mind. Sometimes the colors change, but most stay the same; the number 1 is a bright green color. I do not see the color when I read it on a screen unless I am focusing intently.

2. Numbers also have distinct 'personalities' that do not change. I get a feeling of this 'personality' especially when reading clocks; for example, the number 2 in 2:00 seems reasonable and gentle, but the 9 in 9:00 seems angry and intense.

3. Words can have personalities too, but this seems reserved for the most severe of words or words with the most negative meaning.

4. Some words also carry a sound with them that corresponds to their connotation. For example, when I hear or read the word 'ugly', I get a faint sensation of clanging alarm bells in my ears due to the negative meaning of the word; when I hear or read the word 'grin', I get the sensation of a happy, light buzzing sound.

**9. J.H.**

*Word-colour. Word-gustatory. Orgasm-colour. Music-colour. Misophonia.*

Certain things have colors. Sneezes are a mixture of grey/lavender/blue.

Names have color or smell/taste, or both. My husband's name, "Wes," is blue and it tastes similar to lemons.

The name "Michael" smells/tastes like something bad, akin to the smell of sweaty gym socks. "Colorado" tastes like whipped cream. The name "Sean" is a mustard yellow.

I hope this isn't too personal, but orgasms evoke color for me. Bright, vibrant colors. Similarly, some music provides the same burst of colors.

Not all music gives me bursts of color. Certain songs have a similar response, and I've not been able to pinpoint a rhyme or reason to what type of song - such as genre or instrument.

Also, I have very physical responses to certain touches and sounds, even if I imagine them in my head. I'm not sure if that is part of my synesthesia. Certain songs, again, will give me a physical response: I'll get such intense goose bumps that it becomes a little painful and my body will get tense. The sound of styrofoam squeaking is incredibly painful and makes my mouth fill with saliva. Just writing about it made my mouth start to fill with saliva. It's very odd. I do know that I have misophonia, so maybe it's more related to that? I also believe that the three have to be connected somehow: ASMR, synesthesia and misophonia, because what are the chances that one person would have three separate, unusual conditions such as this? And I've heard from others in the ASMR community that they too experience synesthesia and misophonia. Sometimes I feel that they crossover from one to the other. For instance, if I hear a sound that triggers my miso, sometimes I can concentrate really hard and make it cross over into ASMR.

**10. H.B.K.**

*Pain-gustatory.*

The most frequent synaesthetic experience I have, is whenever I hit my coccyx. Whenever this happens, I taste carrots. The touch (/pain) is always automatically connected with taste. When I come to think of it, the taste of carrots is in fact often related to physical pain in my case.

As of right now, I can't seem to remember any other specific synaesthetic experiences, although I'm sure there are more. I don't write down my experiences, but whenever I experience them, I am not doubting them.

**11. R.R.**

*Music-movement.*

I ofteb listen to music. And I also have very active imagination. And so it lead  for me to visualize music. Most of the time I imagibe it as 3D waves of some liquid or a 2D line. Sometimes even as expanding sound waves. And so when im listening to loud music, increasing tempo this image of a wave starts to raise and becomes more wild like the sea. And so slower music or decreasing sound make me imagibe the waves decreasing and becoming more calm.

**12. S.W.**

*Word-colour. Grapheme-texture(?). Misophonia.*

1. When I hear magazine page flipping, or gum chewing it makes me very lethargic, and often sends me to sleep

2. When I whenever I think of capital letters (i.e ABCDE etc) I imagine making the letters up with staples, so in different sections to make up the letter. Odd, I know, but have done this since school. I've never actually told anyone this!

3. I think of different countries as colours, i.e England - blue, India - yellow, Ireland - green, France - blue, Spain - red, etc. It just springs to mind if the name of the country is mentioned

4. I love the sound of different words and sounds. They've given me 'tingles' in my scalp since I remember. For example, I love the word 'sponge' - it's so relaxing. I also love the noise made when my son plays with his wooden blocks - again, so relaxing.

Other noises disgust me, such as someone talking with a dry mouth - it gives me the shivers

**13. L.P.**

*Grapheme-gender. Word-gender. Grapheme-colour.*

I associate gender with a lot of things:

1 is male, 2 is female, three is female, etc.

The word "basket" is female, "box" is male.

"A" is male, "b" is female, "c" is female.

I also see colors with letters and numbers. "A" is red, "b" blue, "c" yellow, etc.

1 is blue, 2 is red, 3 is purple, etc.

**14. H.A.**

*Grapheme –colour. Grapheme-space. Number-colour. Number-space.*

1) I perceive different numbers as different colors (for example, one that comes to mind is zero is a reddish brown color).

2) I perceive different letters as different colors (for example, the letter e is usually green).

3) I perceive the alphabet as a line of letters on a plane that I see diagonally (far away on the left to close on my right).

4) I perceive numbers on a line not unlike the alphabet except I see it as close on my left and far on my right (and because the number line is infinite, I can see the line go off into infinite space).

It is hard for me to pinpoint a color for each number/letter on purpose. I usually see the color for an instant when I see or think of a letter/number and it is gone if I think about it too hard. I do know, however, that I perceive a color for each number/letter. In regards to the lines of numbers/letters, the space between numbers/letters varies. For example, I perceive the number 70 to be closer to 60 than to 80 and 100 to be closer to 200 than to 0. I also perceive H, I, and J to be relatively close to each other compared to other letters in the alphabet.

**15. A.T.**

*Grapheme-colour. Grapheme-gender. Time-space. Word-texture.*

Letters and numbers have colors (A is pink, B is blue, 1 is orange-yellow, 2 is blue, etc).

Letters and numbers have genders, ages, and personalities. (3 is a mischievous young boy; 5 is a vivacious, bossy teenage or twentysomething female).

I can easily visual time (and where events are placed in relation to time) in a physical space in my mind.

Words sometimes have textures that I feel in my mouth when I say them, and sometimes when others say them.

I learned the alphabet and read at a very young age and I remember being annoyed that my alphabet fridge magnets were not the "right" colors! It's probably also worth mentioning that I started studying Japanese at age 11, and for the hiragana and katakana syllabaries I experience colors, genders, and personalities for each of the symbols, too. (But not as strongly with kanji for some reason?).

The word-texture connection is not as strong as the letter/number - color/gender/personality connection for me, but for example the word "joke" has the texture of a dry chocolate cookie (like an Oreo) and "mat" feels like I'm sinking my teeth into something spongy. Not all words have a texture, but some have a very memorable one.

**16. M.Y.**

*Word-colour. Grapheme-colour.*

I see the word food as a rusty orange colour

I see number 1 as red

I feel banging in my chest when I listen to loud music

I see the word water as lilac

**17. A.B.**

*Sound-movement.*

What I experience is when I hear a sound it is like a movement as well - so like a sideways movement or whatever. I think this is fairly normal but not sure?

**18. J.J.**

*Texture-taste. Sound-colour. Sound-taste. Calendar-shape. Colour-form. Colour-pain(?). Sound-touch.*

1.) I taste certain textures. Ceramics, terra cotta, frosted glass- my mouth becomes chalky and dry.

2.) Certain notes or chords have color and minimal image. Image is hard to explain, but for color- Am is brick red, Em is gray, C is yellow, G is green, etc.

3.) Certain pitches taste metallic.

4.) Certain things like lists or the calendar year can be twisted or circular in shape and have certain colors at certain points.

5.) Sometimes colors have volume, or can be painful to look at.

6.) Certain sounds can feel prickly, or tickly or can make my mouth water.

**19. B.Z.**

*Grapheme-colour. Number-colour.*

I see the letter A as yellow
I see the letter B as brown

I see number 5 as red

I see number 6 as orange

I see all the numbers and letters in colour.

**20. A.W.**

*Number-personality.*

For me, numbers (and sometimes letters) appear to have a sort of personality. For instance, number 5 has different personality traits than 3. It comes off more stern, while 3 is somewhat... Softer. I feel it if I'm doing math or simply just writing a phone number. It is difficult for me to explain but I can try to elaborate in full for each number if you would like.

**21. D.K.**

*Music-movement. Sound-colour.*

I suffer chronic pain due to disability, normally i can associate colour with everything, but when asked by a pain specialist to describe my pain by colour, i was stumped. And i still cannot give it a colour.

When i hear loud orchestral style " movie drama " type noise, such as at cinema i feel upward sensations.

I have a colour sensation with animal sounds, mostly all are white!

Hope this helps, as i remember more and notice new ones i would be happy to forward them to you. Right now i am drawing blank yet i experience constantly.

**#22 J.H.**

*Calendar-space.*

My synaesthesia really only manifests in one way, and that is in the representation of the calendar year and the months/days within it, which form a pictorial timeline in my head, that I use daily.  For example, if someone mentions a date in history, I will see the corresponding place on the timeline, surrounded by the shape of dates either side of it, and am able then to draw images from its general area corresponding to my understanding of that period.

**23. G.D.O.**

*Number-colour. Word-colour. Music-form. Person-colour(?)*

Below is my list of four things I experience as synesthesia. It was rather difficult for me to make this list. I think that I experience synesthesia in other ways as well, but this is such a normal way of experiencing the world for me, that I am not even aware of it.

1) I 'see' the number one on a dice as yellow

2) I 'see' Sunday as purple

3) I 'see' moving, coloured, and textured patterns when I listen to music (very detailed, and depending completely on the specific song)

4) I 'see' my philosophy teacher as a blueish green

**24. J.B.**

*Number-colour. Music-colour.*

The strongest form of synaesthesia I have is with numbers.*

I also associate colors to different genres of music.**

Both of these are not very strong and they only work one way. Also not everything has a color. Or a lot of numbers in sequence have the same color. I think I have read something about people who would also be able add two numbers and get the mixed color (i.e. 3=red, 2=blue, 5=purple), but that is something I certainly don't experience.

The music genres are probably more of an association I have because of certain bands or instruments, so I'm not entirely sure that is really a form of synaesthesia.

Other than that I can't think of any other forms of synaesthesia that I experience. I hope this will help you with your study.

*For example, I associate the number 21 with yellow, 16 with red, 3 with green. Larger numbers are often a color of which one number is already in the large number (2 is yellow, almost everything in the 20's is yellowish).

**For example, Classical music is yellow/gold (probably because of the wind-instruments), Metal is blue, pop is a light red, gothic is a dark red.

**25. J.L.**

*Music-form.*

When I listen to music it resembles shapes or motions and sometimes colors in my head but I don't actually see things.

I'm not sure if that's real synaesthesia though because I don't know if synaesthesia means associations or "overlapping stimuli", for example actually seeing colors or shapes when hearing music.

For me it's more like I hear the music and most of the time I couldn't draw the shapes or movements it resembles in my head but it's not like stuff is going on before my eyes, it's all in my head.

**26. L.M.**

*Music-colour.*

I mainly experience synaesthesia through music. When I hear slower, more somber music, I see blue. Faster, more angry sounding music brings red, and sharp, high music I will see yellow. Let me know if you need any more information, thanks!

**27. F.B.**

*Number-colour. Day-colour. Action-colour(?)*

My three examples for my strongest synaesthesia:

1) I see the number 3 as red, 4 as green, 5 as blue, 6 as yellow.

2) I see tuesday as yellow, wednesday as green, thursday as blue.

3) Childhood memories: I see beeing on my way to school as green, or helping my mother in the kitchen as yellow.

**28. A.S.**

*Colour and number synaesthetic associations.*

Seeing red made me uncomfortable once

       - I had a class schedule typed in black font. Five classes listed vertically. One class wasn't my first option and when placed on the list, it made my entire schedule "red". I didn't like the way that one class made the rest of my classes look.

Working out

       - When I exercise, I count in my head and see the numbers behind colorful backgrounds. I think 34 or 38 was orange.

Turning four years old

       - When I was four, I imagined a 4 in front of celestial blue background. Rainbow colors were traveling in the 4 like a train. The 4 would move back and forth with a voice saying 4 every time the 4 moved forward

Scissors

         -I watch MinxLaura123's ASMR channel on YouTube. She uses two pairs of scissors when conducting haircut role plays. Her first pair sound like a line (I mean the math term) while the second pair sound like a ray (the math term)

**S.T.**

***No synaesthesia***

Sorry for taking so long to reply, I don't check this e-mail address very often. Unfortunately, I can't answer your question because I don't get synesthesia. I'm not sure why I would have said I do, perhaps the question was phrased in a way that lead me to answer in the positive without being explictily about synaesthesia. If the question was 'Do you get synaesthesia?' I doubt I would have answered 'Yes'. I may have be talking about some kind of associative effect rather than synaesthesia directly.

**F.v.S.**

***No synaesthesia***

I did take part in that study, but I'm not sure which answer of mine sounded like synaesthesia, so I'm not sure how tp answer your questions. Feel free to elaborate or ask more questions though, I'm always glad to be of help.

**A.F.**

***No synaesthesia***

My apologies. I after researching the characteristics of synethasia. I realize that I do not have it. I read the descriptor for it and misunderstood it to include visualization. I do not experience this phenomena outside of myself but only through meditation and daydreams. I apologize for taking your time and wish you best of luck as a fellow researcher.

**L.C.**

***No synaesthesia – cross-sensory interactions.***

My synesthesia is presented as more distortions of other senses when one is being overwhelmed.

For example, when I’m experiencing ASMR, my hearing will become foggy or distorted.

Similarly, if I’m standing and experience ASMR, my vision will become blurry and I’ll feel light headed as though I need to pass out.

In college I was plagued with terrible migraines that presented with aura. While lying down to get over the migraine I would frequently feel as though I was nearly paralyzed- when my phone rang it took nearly 10 minutes to “convince” my arm to get it. I found relief first in beta blockers but now find preventative relief with ASMR use daily. I’m certain this all boils down to blood pressure issues in my brain; my body has a very low blood pressure but when I have migraines it spikes the blood pressure in my brain. When the migraine is relieved I’ll feel as though my ears are steaming while the blood re-distributes itself elsewhere in my body.

**B.C.**

***Two single instances of a synaesthetic association, otherwise hallucinatory.***

I hear certain animated gifs even though they are obviously silent (pretty common I think?)  

I feel like the number 4 is blue, 7 is yellow.

I don't know if this is revelent, but I've always experienced what I call "phantom tv sounds".  This is where I'll be busy doing something and be able to hear the faint sounds of a TV show coming from another room - but when you go into the room and see the TV is off, the sounds stop.  This doesn't happen to me as often anymore as it did when I was young.

**G.D.S.**

***No synaesthesia. Mild hallucinatory olfactory experiences.***

I often smell when someone mentions something, for example, if someone mentions an item, I might then sense that I smell that.  It does not occur frequently, is not long lasting, and it is very subtle.

**L.T.**

***No synaesthesia***

1. Sometimes I will imagine particular landscapes and images when I listen to certain music - these are not places I have not physically been to before i.e. not a memory. Like when I listen to Coldplay or Travis, I’ll often imagine an English countryside, daffodils, daisies, green grass, seaside - depends on how I’l feeling as well. When I feel sad, the images are usually in the rain, or on an overcast/grey day.

2. Sometimes, when my eyes are closed, I’ll see coloured patterns flash and fade on a black background - similar to a screensaver. The patterns are like a kaleidoscope, and are usually circles or spirals moving and overlapping.

3. And sometimes when I listen to music, I see the words/lyrics in my head - usually black background, and the words are in white. Each word will flash, and quickly fade in time for the new words.
